# Protection of the Extracts of *Lentinus edodes* Mycelia against Carbon-Tetrachloride-Induced Hepatic Injury in Rats

**DOI:** 10.1100/2012/231586

**Published:** 2012-05-22

**Authors:** Mei-Fen Chen, Hsien-Hui Chung, Han-Lin Lu

**Affiliations:** ^1^Department of Nursing and Department of Biological Science and Technology, Chung Hwa University of Medical Technology, Jen-Te, Tainan City 71703, Taiwan; ^2^Institute of Basic Medical Sciences, College of Medicine, National Cheng Kung University, Tainan City 70101, Taiwan; ^3^Department of Chinese Medicine, St. Joseph Hospital, Lin-Yah, Kaohsiung City 80288, Taiwan

## Abstract

*Lentinus edodes* is the medicinal macrofungus showing potential for therapeutic applications in infectious disorders including hepatitis. In an attempt to develop the agent for handling hepatic injury, we used the extracts of *Lentinus edodes* mycelia (LEM) to screen the effect on hepatic injury in rats induced by carbon tetrachloride (CCl_4_). Intraperitoneal administration of CCl_4_ not only increased plasma glutamic oxaloacetic transaminase (GOT) and glutamic pyruvic transaminase (GPT) but also decreased hepatic superoxide dismutase (SOD) and glutathione peroxidase (GPx) levels in rats. Similar to the positive control silymarin, oral administration (three times daily) of this product (LEM) for 8 weeks significantly reduced plasma GOT and GPT. Also, the activities of antioxidant enzymes of SOD and GPx were elevated by LEM. in liver from CCl_4_-treated rats, indicating that mycelium can increase antioxidant-like activity. Moreover, the hepatic mRNA and protein levels of SOD and GPx were both markedly raised by LEM. The obtained results suggest that oral administration of the extracts of *Lentinus edodes* mycelia (LEM) has the protective effect against CCl_4_-induced hepatic injury in rats, mainly due to an increase in antioxidant-like action.

## 1. Introduction

Hepatic injury may result from many risk factors, such as hepatic virus, inflammation, and alcohol consumption [1–3]. Oxidative stress is a state of redox imbalance resulted from elevated reactive oxygen species (ROS) generation and decreased antioxidant capacity [4]. In general, obesity is believed as an important risk factor for liver injury in man [5]. Carbon tetrachloride- (CCl_4_-) induced hepatic injury is a well-established animal model due to generation of oxidative stress to result in hepatic oxidative damage and inflammation [6]. Thus, CCl_4_-induced hepatic injury is a common animal model widely used to evaluate the hepatoprotective action of testing compounds [7–9].

*Lentinus edodes* is the medicinal macrofungus showing potential for therapeutic applications in infectious disorders including hepatitis [10]. The extract of *Lentinus edodes* Mycelia (LEM) exhibits medicinal effects such as anticancer activity and immunoregulatory activity [11]. Protection of LEM against liver damage induced by chemicals has been documented such as dimethylnitrosamine [12] and D-galactosamine [13]. Both reports were carried out in cultured hepatic cells. However, the effect of LEM on hepatic injury in animal remains unclear. Thus, we screen the extracts of LEM in rats using CCl_4_-induced liver damage model while silymarin is used as a positive control because it is popularly applied as the protective substance in CCl_4_-induced hepatic damage [14, 15]. The main aim of the present study is going to clarify the role of antioxidant-like activity in the liver protection of this product (LEM). 

## 2. Materials and Methods

### 2.1. Materials

The LEM powder named as *Shung-Kang* in Chinese was kindly supplied from Tsung Hsin Tsan Company (Neihu Technology Research Institute, Taipei City, Taiwan). It was prepared as previously reported [16]. Briefly, *Lentinus edodes *mycelia were cultivated in a solid medium composed of sugar-cane bagasse and defatted rice bran. The medium with mycelia was incubated for digestion with mycelial enzymes in water at 30–55°C, and then the incubation temperature was increased to 90°C for inactivation of the enzymes and sterilization. The digest was filtered, lyophilized, and used as the LEM preparation. Moreover, the anti-rat SOD and GPx antibodies to measure hepatic SOD and GPx proteins were purchased from Biodesign (Saco, ME, USA). The plasma glutamic oxaloacetic transaminase (GOT) and glutamic pyruvic transaminase (GPT) were measured by assay kits from AppliedBio (Hercules, CA, USA). In addition, the activities of hepatic SOD and GPx were determined using the kits from Cayman Chemical Company, Inc. (Ann Arbor, Michigan, USA). Silymarin alone or LEM alone did not alter liver injury parameters by themselves (data not shown).

### 2.2. Experimental Animals

Male Wistar rats aged 8 weeks were obtained from the Animal Center of National Cheng Kung University Medical College. They were maintained in a temperature-controlled room (25 ± 1°C) and kept on a 12 : 12 light-dark cycle (light on at 06:00 h). Food and water were available *ad libitum*. All animal procedures were performed according to the Guide for the Care and Use of Laboratory Animals of the National Institutes of Health as well as the guidelines of the Animal Welfare Act.

### 2.3. Experimental Protocols

#### 2.3.1. Chronic Administration of LEM in CCl_**4**_-Treated Rats

The dried LEM powder was dissolved in saline solution for oral administration at the desired doses. The hepatic injury of rats was induced with CCl_4_ (1 mL/kg, i.p.) dissolved in olive oil. In the present study, all experimental rats were divided into six groups: (1) vehicle-treated Wistar rats, (2) vehicle-treated CCl_4_-induced rats, (3) silymarin (200 mg/kg)-treated CCl_4_-induced rats, (4) LEM (100 mg/kg)-treated CCl_4_-induced rats, (5) LEM (200 mg/kg)-treated CCl_4_-induced rats, and (6) LEM (500 mg/kg)-treated CCl_4_-induced rats. In brief, CCl_4_-induced rats received an oral administration of silymarin or LEM at the indicated dose or the same volume of vehicle three times daily for 8 weeks. Blood samples (1 mL) of the treated rats were collected under sodium pentobarbital anesthesia (30 mg/kg, i.p.) from the femoral vein at the indicated time point for measurement of plasma glutamic oxaloacetic transaminase (GOT) and glutamic pyruvic transaminase (GPT) using an autoanalyzer (Quik-Lab, Ames, Miles Inc., Elkhart, Indiana, USA).

#### 2.3.2. Determination of Mn-SOD and Cu/Zn-SOD Activities

Rats were sacrificed under sodium pentobarbital anesthesia (60 mg/kg, i.p.). The liver was removed and we washed liver tissues with saline to remove as much blood as possible. Mn-SOD and Cu/Zn-SOD concentrations were determined using commercially available rat assay kits from Cayman Chemical Company, Inc. (Ann Arbor, MI, USA). Hepatic samples were homogenized at 4°C in ice-cold homogenized buffer containing 0.32 mol/L Sucrose, 1 mmol/L EDTA, and 10 nmol/L Tris-HCl, pH 7.4 in a Teflon/glass homogenizer. The homogenate was centrifuged at 1400 ×g for 5 min at 4°C and the supernatant was centrifuged at 4500 ×g for 10 min. Then, the supernatant was centrifuged at 11000 ×g for 60 min. We analyzed the supernatant to measure Cu/Zn-SOD activity, and the pellet was used to measure Mn-SOD activity. Then, we added 20 *μ*L of sample, 200 *μ*L of reagent solution, and 20 *μ*L of enzyme working solution to the wells and mixed thoroughly. We incubated the plate at 37°C for 20 min. The determination of Mn-SOD and Cu/Zn-SOD in samples was carried out and the absorbance was measured by a SPECTRAmax 340PC ELISA reader (Molecular Devices Corporation, Union City, CA, USA) at 450 nm. SOD activity was expressed as U/mg protein. 

#### 2.3.3. Determination of GPx Activity

Rats were sacrificed under sodium pentobarbital anesthesia (60 mg/kg, i.p.). The liver was removed and GPx concentration was determined using a commercially available assay kit from Cayman Chemical Company, Inc. (Ann Arbor, Michigan, USA). Hepatic samples were homogenized at 4°C in ice-cold homogenized buffer containing 50 mmol/L Tris-HCl, 5 mmol/L EDTA, and 1 mmol/L DTT, pH 7.5 in a Teflon/glass homogenizer. The homogenate was centrifuged at 10000 ×g for 15 min at 4°C and the supernatant was used for GPx quantification. Then, we added 20 *μ*L of sample, 100 *μ*L of assay buffer and 50 *μ*L cosubstrate mixture to the wells. To initiate the reactions, we added 20 *μ*L of cumene hydroperoxide to the wells being used and carefully shook the plate for a few seconds to mix. The determination of GPx in samples was carried out and the absorbance was measured by an SPECTRAmax 340PC ELISA reader (Molecular Devices Corporation, Union City, CA, USA) at 340 nm. GPx activity was expressed as nmol NADPH/min/mg protein.

#### 2.3.4. Northern Blotting Analysis of mRNA Level

Northern blotting analysis was obtained from four individual experiments. Total RNA was extracted from the liver of all experimental animals using the UltraspecTM-II RNA extraction system. The concentration of RNA was measured using the absorbance at 260 nm. For Northern blotting analysis, total RNA (40 *μ*g) was denatured in a solution containing 2.2 mmol/L formaldehyde and 50% formamide (v/v) by heating at 55°C for 15 min. Aliquots of total RNA were then size-fractionated in a 1.2% agarose/formaldehyde gel. Ethidium bromide staining was used to identify the position of the 18S and 28S rRNA subunits and to confirm that equivalent amounts of undegraded RNA had been loaded. The fractionated RNA was transferred to a hybond-N membrane (Amersham Corp., Bucks, UK) and crosslinked by UV irradiation (Stratagene, CA, USA). Probes were labeled with [*γ*-32P] dCTP (New England Nuclear, Boston, USA) using the Medaprime labeling system kit (Amersham Corp., Bucks, UK). Hybridizations were carried out in medium containing denatured salmon sperm DNA (100 *μ*g/mL) at 65°C for 2 hrs. The membrane was washed twice for 20 min in 2 × sodium saline citrate (SSC)/0.1% SDS at room temperature and once for 20 min in 0.1 × SSC/0.1% SDS at 40°C. Autoradiograms were prepared on Kodak X-ray (Rochester, NY, USA) film using a single enhancing screen at −80°C. Intensities of the mRNA bands on the blot were quantified by scanning densitometry (Hoefer, San Francisco, CA, USA). The response of *β*-actin was used as an internal standard.

#### 2.3.5. Western Blotting Analysis of Protein Level

Western blotting analysis was obtained from four individual experiments. After homogenization of the liver from all experimental rats using a glass/Teflon homogenizer, the homogenates (50 *μ*g) were separated by sodium dodecyl sulfate-polyacrylamide gel electrophoresis, and Western blot analysis was performed using an anti-rat Mn-SOD antibody, Cu/Zn-SOD antibody, and GPx antibody (1 : 1000) purchased from Biodesign (Saco, ME, USA) in liver. The blots were probed with a goat polyclonal actin antibody (1 : 500) from Zymed Laboratories (South San Francisco, CA, USA) to ensure that the amount of protein loaded into each lane of the gel was constant. Blots were incubated with the appropriate peroxidase-conjugated secondary antibodies. After removal of the secondary antibodies, the blots were washed and developed using the ECL-Western blotting system. Densities of the obtained immunoblots at 25 KDa for Mn-SOD, 16 KDa for Cu/Zn-SOD, 24 KDa for GPx, and 43 KDa for actin were quantified using laser densitometer.

#### 2.3.6. Histological Analysis

The liver tissues were removed from each group of rats and fixed in 10% formaldehyde at 4°C for 2 days. Fixed specimens were dehydrated and embedded in paraffin. The specimens were then cut into 5 *μ*m thick sections at 50 *μ*m intervals and then stained with hematoxylin and eosin (H&E; Muto Pure Chemicals, Tokyo, Japan). The sections were then observed with a light microscope. 

#### 2.3.7. Data Analysis

The results are expressed as the standard error of the mean (S.E.M.) from the number (*n*) of individual experiments performed. Statistical analysis was performed by one-way ANOVA followed by Dunnett's post hoc test. A probability level of *P* value less than 0.05 was required for statistical significance.

## 3. Results

### 3.1. Effect of LEM Administration on Liver Damage in CCl_**4**_-Treated Rats

**Figure 1:**
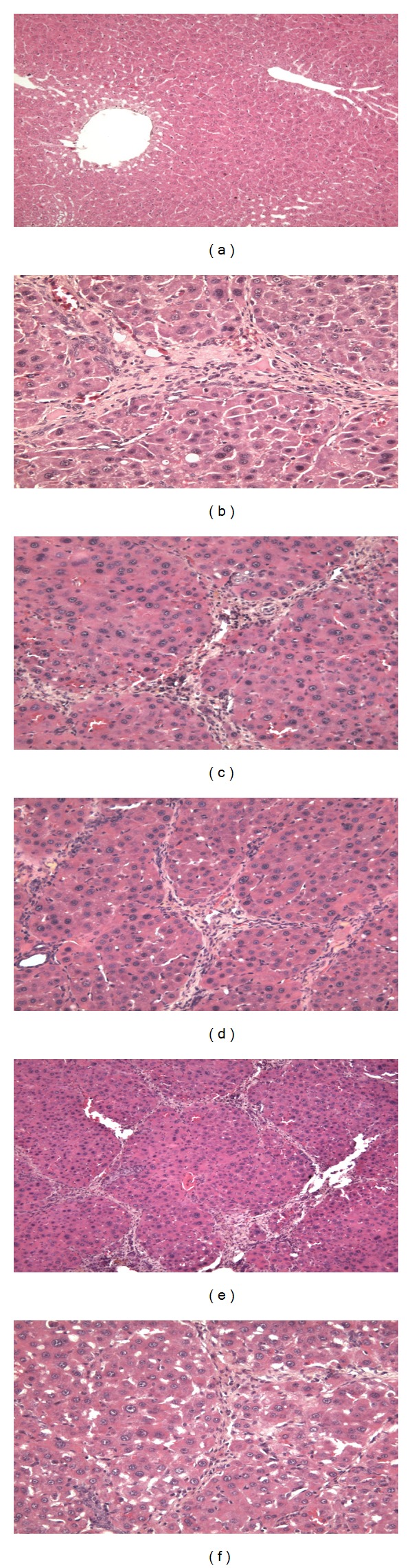
Effect of LEM on hepatic injury in CCl_4_-treated rats. All rats were received oral administration at indicated dose three times per day. (a) Normal rats treated with vehicle. (b) Normal rats treated with CCl_4_ for 8 weeks. (c) CCl_4_-treated rats received oral administration of silymarin (200 mg/kg) for 8 weeks. (d) CCl_4_-treated rats received oral administration of LEM (100 mg/kg) for 8 weeks. (e) CCl_4_-treated rats received oral administration of LEM (200 mg/kg) for 8 weeks. (f) CCl_4_-treated rats received oral administration of LEM (500 mg/kg) for 8 weeks. Histology of liver was characterized by staining with hematoxylin-eosin (Magnification: ×400).

Rats treated by CCl_4_ significantly induced hepatic injury. As shown in Figure 1(b), inflammatory cells were observed in CCl_4_-induced hepatic tissues. Oral administration of 200 mg/kg silymarin for 8 weeks improved CCl_4_-induced hepatic injury markedly (Figure 1(c)). In addition, oral administration of LEM for 8 weeks also markedly improved CCl_4_-induced hepatic injury in a dose-dependent manner (Figures 1(d), 1(e), and 1(f)).

### 3.2. Effect of LEM on Plasma Glutamic Oxaloacetic Transaminase (GOT) and Glutamic Pyruvic Transaminase (GPT) Levels in CCl_**4**_-Treated Rats

**Figure 2:**
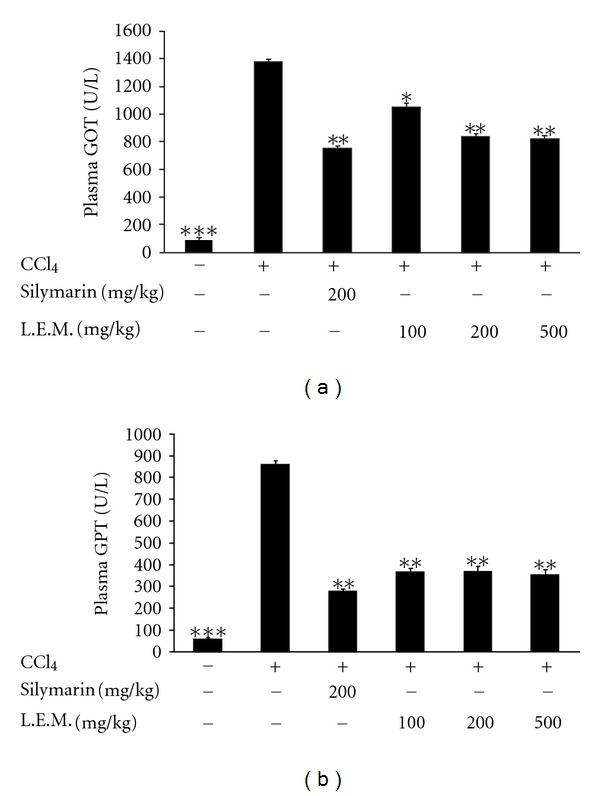
Effect of LEM on plasma GOT and GPT levels in CCl_4_-treated rats. (a) Changes of plasma GOT by oral administration of LEM in CCl_4_-treated rats for 8 weeks. (b) Changes of plasma GPT by oral administration of LEM in CCl_4_-treated rats for 8 weeks. Lane 1: vehicle-treated Wistar rats; lane 2: vehicle-treated CCl_4_-induced rats; lane 3: silymarin- (200 mg/kg) treated CCl_4_-induced rats; lane 4: LEM- (100 mg/kg) treated CCl_4_-induced rats; lane 5: LEM- (200 mg/kg) treated CCl_4_-induced rats; lane 6: LEM- (500 mg/kg) treated CCl_4_-induced rats. Data represent mean ± SEM of eight animals. *P < 0.05, **P < 0.01, and ***P < 0.001 compared with CCl_4_-induced group receiving vehicle.

As shown in Figure 2(a), CCl_4_-treated rats showed a higher plasma GOT level as compared with vehicle-treated normal rats. Similar to the rats treated with silymarin (200 mg/kg) for 8 weeks as positive control, oral administration of LEM into CCl_4_-treated rats three times daily for 8 weeks significantly lowered the plasma GOT level (100 mg/kg, 200 mg/kg, and 500 mg/kg). Same results were also observed in plasma GPT levels as shown in Figure 2(b).

### 3.3. Effect of LEM on the Activities of Hepatic Superoxide Dismutase (SOD) and Glutathione Peroxidase (GPx) in CCl_**4**_-Induced Rats

**Figure 3:**
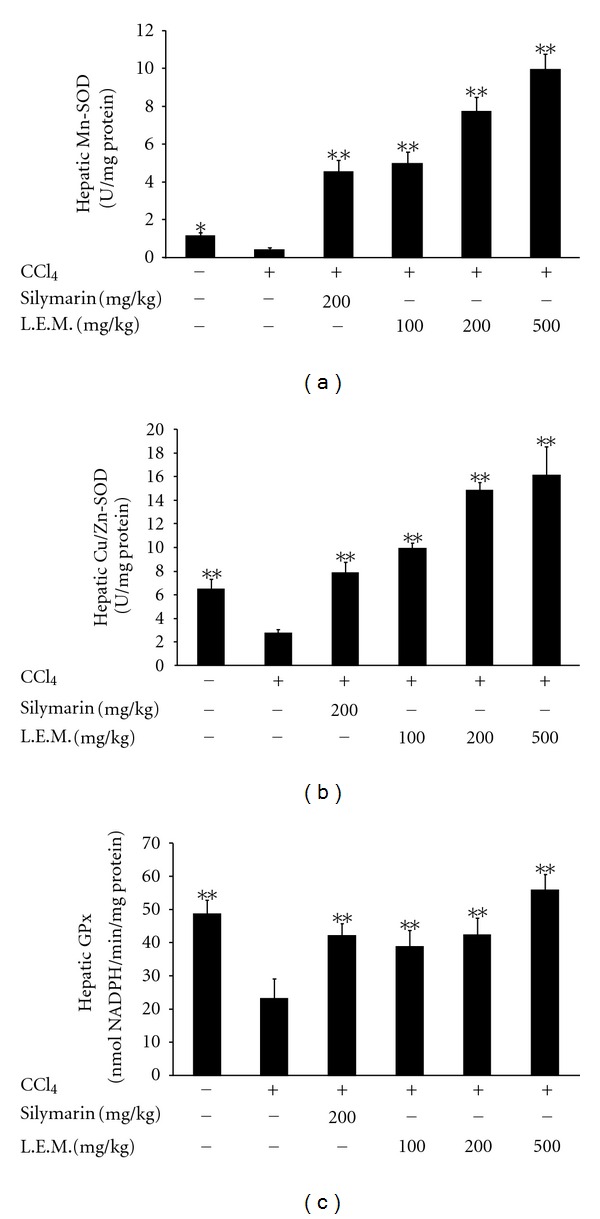
Effects of LEM on activities of hepatic Mn-SOD, Cu/Zn-SOD, and glutathione peroxidase (GPx) in CCl_4_-treated rats. (a) Changes of hepatic Mn-SOD by oral administration of LEM in CCl_4_-treated rats for 8 weeks. (b) Changes of hepatic Cu/Zn-SOD by oral administration of LEM in CCl_4_-treated rats for 8 weeks. (c) Changes of hepatic GPx by oral administration of LEM in CCl_4_-treated rats for 8 weeks. Lane 1: vehicle-treated Wistar rats; lane 2: vehicle-treated CCl_4_-induced rats; lane 3: silymarin- (200 mg/kg) treated CCl_4_-induced rats; lane 4: LEM- (100 mg/kg) treated CCl_4_-induced rats; lane 5: LEM- (200 mg/kg) treated CCl_4_-induced rats; lane 6: LEM- (500 mg/kg) treated CCl_4_-induced rats. Data represent mean ± SEM of eight animals. * *P* < 0.05 and ** *P* < 0.01 compared with CCl_4_-induced group receiving vehicle.

CCl_4_-treated rats showed lower levels of both Mn-SOD and Cu/Zn-SOD in liver as compared with vehicle-treated normal rats. Oral administration of silymarin (200 mg/kg) three times daily for 8 weeks produced a significant elevation in the activities of hepatic Mn-SOD and Cu/Zn-SOD. Also, oral administration of LEM into CCl_4_-treated rats in same manner resulted in an increase of Mn-SOD and Cu/Zn-SOD activities in liver (Figures 3(a) and 3(b)). As shown in Figure 3(c), CCl_4_-treated rats showed a lower activity of GPx in liver than the vehicle-treated normal rats. Similar to the effect of silymarin (200 mg/kg), oral administration of LEM into CCl_4_-treated rats three times daily for 8 weeks also produced an increase of GPx activity in liver.

### 3.4. Effects of LEM on Gene Expressions of Antioxidative Enzymes in Liver of CCl_**4**_-Treated Rats

**Figure 4:**
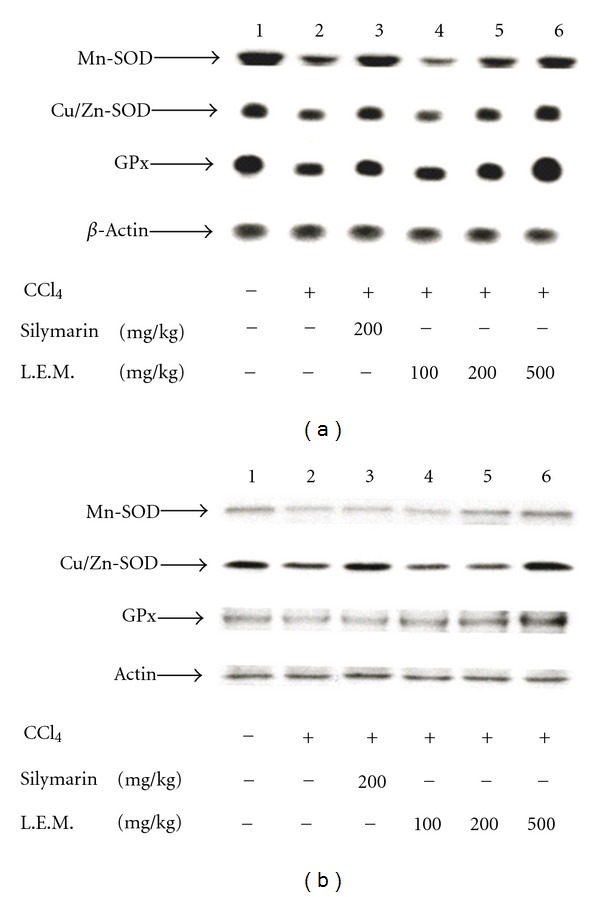
Effects of LEM on gene expressions of Mn-SOD, Cu/Zn-SOD, and glutathione peroxidase (GPx) in liver of CCl_4_-treated rats. (a) The representative picture showing mRNA levels for Mn-SOD, Cu/Zn-SOD, GPx, or *β*-actin in liver isolated from CCl_4_-induced rats receiving treatment with silymarin or LEM three times daily for 8 weeks. Lane 1: vehicle-treated Wistar rats; lane 2: vehicle-treated CCl_4_-induced rats; lane 3: silymarin- (200 mg/kg) treated CCl_4_-induced rats; lane 4: LEM- (100 mg/kg) treated CCl_4_-induced rats; lane 5: LEM- (200 mg/kg) treated CCl_4_-induced rats; lane 6: LEM- (500 mg/kg) treated CCl_4_-induced rats. (b) The representative response of protein level for Mn-SOD, Cu/Zn-SOD, GPx, or actin in liver isolated from CCl_4_-induced rats receiving treatment with silymarin or LEM three times daily for 8 weeks. All lanes are expressed as the same as mRNA level (a) on the above.

CCl_4_-treated rats showed a decrease in mRNA levels of Mn-SOD, Cu/Zn-SOD, and GPx in liver. Oral administration of LEM into CCl_4_-treated rats three times daily for 8 weeks produced an elevation in mRNA levels of Mn-SOD, Cu/Zn-SOD, and GPx in liver (Figure 4(a)). Western blotting analysis also showed a similar reduction on the protein levels of Mn-SOD, Cu/Zn-SOD, and GPx in liver of CCl_4_-treated rats. Similarly, elevation in protein levels of Mn-SOD, Cu/Zn-SOD, and GPx was produced by LEM in a dose-dependent manner (Figure 4(b)). The data for quantification of Mn-SOD, Cu/Zn-SOD, and GPx mRNA values were shown in Table 1. Also, the data for quantification of Mn-SOD, Cu/Zn-SOD, and GPx protein values were presented in Table 2.

## 4. Discussion

In the present study, we found that the extracts of *Lentinus edodes *mycelia (LEM) can significantly lower plasma GOT and GPT in CCl_4_-treated rats. Also, histological change of liver induced by CCl_4_ was improved by treatment with LEM for 8 weeks. Moreover, LEM. significantly increased the activities of hepatic Mn-SOD, Cu/Zn-SOD, and GPx after 8 weeks of treatment. Therefore, improvement of CCl_4_-induced hepatic injury by LEM seems to be related to an increase in antioxidant-like activities.

Plasma GOT and GPT levels are reliable makers for the evaluation of hepatic injury. CCl_4_-induced hepatic injury showed a marked increase in plasma GOT and GPT levels as described previously [17, 18]. Also, as shown in Figure 1, histological change of liver was observed in rats that received CCl_4_ showing the success of this animal model. Similar to previous reports [19, 20], silymarin significantly decreased the plasma GOT and GPT levels in CCl_4_-treated rats. In addition, LEM also lowered the plasma GOT and GPT levels in CCl_4_-treated rats and improved the hepatic injury (Figure 1). The protective effect of LEM against CCl_4_-induced liver damage can thus be considered in rats. This is consistent with the previous reports using dimethylnitrosamine [12] or D-galactosamine [13].

Hepatic injury induced by CCl_4_ was associated with oxidative stress due to CCl_4_-induced free radical production [21, 22]. In addition, inflammatory cytokines TNF-*α* and IL-6 were also involved in this hepatic injury while the antioxidative compound (resveratrol) can protect hepatic injury by suppressing oxidative stress [23]. Thus, we focused on the change of oxidative stress in this action of LEM. In general, superoxide dismutase (SOD) is an important antioxidant enzyme which catalyzes the conversion of toxic superoxide radical to less reactive hydrogen peroxide [24]. SOD is known to be reduced markedly in CCl_4_-induced hepatic injury [25] while oxidative stress could be ameliorated via the elevation of hepatic SOD level [26]. In addition, glutathione peroxidase (GPx) is another antioxidant enzyme commonly used to investigate the oxidative stress [27]. It has been indicated that antioxidant-like compounds produce hepatic protection through an increase in GPx to scavenge the free radicals [28, 29]. In the present study, LEM increased the activities of Mn-SOD, Cu/Zn-SOD, and GPx in liver of CCl_4_-treated rats (Figure 3). Similar results were also observed in gene expressions of these enzymes using Western blotting analysis for protein level or Northern blotting analysis for mRNA level (Figure 4). Data of quantification show the markedly changes of gene expressions; both mRNA level (Table 1) and protein level (Table 2) in liver of rats received LEM against CCl_4_ in a way similar to silymarin. Thus, increase of hepatic SOD and GPx levels is related to the decrease of plasma GOT and GPT levels by LEM in rats that received CCl_4_-induced hepatic injury.

The main components of LEM are sugars, proteins, polysaccharides, and polyphenolic compounds. The polysaccharides including lentinan, eritadenine, shiitake mushroom mycelium, and culture media extracts (LEM, LAP, and KS-2) are known to enhance immunity [10] while glycogen-like polysaccharides have macrophage-activating activity [11]. Polyphenols are mentioned to produce protection against cardiovascular disease and neurodegenerative disorders in addition to cancers [30–32]. Among polyphenols, syringic acid and vanillic acid are enriched in the solid medium of cultured LEM [33]. *L. edodes *grown in lignocellulose secretes lignin-degrading peroxidase into the culture medium [34]. The mycelia-derived enzymes degrade the lignin to produce phenolic compounds, particularly syringic acid and vanillic acid, which are introduced as active principles for live protection of LEM [35]. However, more investigations are required to make clear of the real substances for liver protection of LEM in the future.

Basically, LEM is the popular edible mushroom in the global market which is attributed to its nutritional value. Thus, the safety of LEM has been confirmed in clinics [36] in addition to animals [37]. Also, LEM can improve atherosclerosis [38] and produce many pharmacological properties including the antibiotic, anticarcinogenic, and antiviral actions from intracellular fraction (fruiting body and mycelia) and extracellular fraction (culture media) as described previously [10]. The potential of LEM is unquestionable as important one in the applications of medicinal therapy.

In conclusion, from the obtained results, the present study is the first one that provided the evidence that oral administration of the extracts from *Lentinus edodes* mycelia (LEM) improves hepatic injury induced by CCl_4_ in Wistar rats. The merits of LEM from antioxidant-like action which is not mentioned before also make it to be a suitable agent for the treatment of hepatic injury in the future.

## Figures and Tables

**Table 1:** The quantification of mRNA levels for Mn-SOD, Cu/Zn-SOD, GPx, or *β*-actin in liver isolated from CCl_4_-induced rats receiving treatment with silymarin or LEM three times daily for 8 weeks. Quantification of mRNA levels using Mn-SOD/*β*-actin, Cu/Zn-SOD/*β*-actin, and GPx/*β*-actin was expressed as mean with standard error (SE) (*n* = 4 per group) in each group. *P < 0.05 compared with CCl_4_-induced group receiving vehicle.

	Mn-SOD (arbitrary units) mRNA/*β*-actin	Cu/Zn-SOD (arbitrary units) mRNA/*β*-actin	GPx (arbitrary units) mRNA/*β*-actin
Control	1.31 ± 0.11*	1.20 ± 0.08*	1.91 ± 0.17*
CCl_4_	0.69 ± 0.06	0.75 ± 0.05	1.03 ± 0.08
+ Silymarin			
200 mg/kg	1.15 ± 0.07*	1.37 ± 0.07*	1.48 ± 0.11*
+ LEM			
100 mg/kg	0.64 ± 0.04	1.24 ± 0.09*	1.50 ± 0.18*
200 mg/kg	0.82 ± 0.05*	1.37 ± 0.11*	1.55 ± 0.13*
500 mg/kg	1.09 ± 0.08*	1.71 ± 0.13*	2.10 ± 0.15*

**Table 2:** The quantification of protein levels for Mn-SOD, Cu/Zn-SOD, GPx, or actin in liver isolated from CCl_4_-induced rats receiving treatment with silymarin or LEM three times daily for 8 weeks. Quantification of protein levels using Mn-SOD/actin, Cu/Zn-SOD/actin, and GPx/actin was expressed as mean with standard error (SE) (*n* = 4 per group) in each group. *P < 0.05 compared with CCl_4_-induced group receiving vehicle.

	Mn-SOD (arbitrary units) protein/actin	Cu/Zn-SOD (arbitrary units) protein/actin	GPx (arbitrary units) protein/actin
Control	1.24 ± 0.07*	1.42 ± 0.15*	1.18 ± 0.08*
CCl_4_	1.01 ± 0.04	1.02 ± 0.05	0.82 ± 0.06
+ Silymarin			
200 mg/kg	1.16 ± 0.05*	1.60 ± 0.09*	1.11 ± 0.05*
+ LEM			
100 mg/kg	0.82 ± 0.06	0.91 ± 0.04	1.06 ± 0.05*
200 mg/kg	1.03 ± 0.11	1.07 ± 0.03	1.23 ± 0.14*
500 mg/kg	1.21 ± 0.10*	1.67 ± 0.06*	1.59 ± 0.17*
